# Impact of using a broad-based multi-institutional approach to build capacity for non-communicable disease research in Thailand

**DOI:** 10.1186/s12961-019-0464-8

**Published:** 2019-06-14

**Authors:** Kathleen Potempa, Benjaporn Rajataramya, Debra L. Barton, Naruemol Singha-Dong, Rob Stephenson, Ellen M. L. Smith, Matthew Davis, Ivo Dinov, Benjamin M. Hampstead, James E. Aikens, Laura Saslow, Philip Furspan, Atiya Sarakshetrin, Srijan Pupjain

**Affiliations:** 1 School of Nursing, University of Michigan, 400 N. Ingalls, Ann Arbor, MI 48109-5482 United States of America; 2 Praboromarajchanok Institute for Health Workforce Development, Ministry of Public Health, Nonthaburi, 110000 Thailand; 3 Department of Research, Institute of Nursing, Suranaree University of Technology, Nakhon Ratchasima, Thailand; 4 Department of Psychiatry-Neuropsychology, University of Michigan, 2101 Commonwealth, Ste C, Ann Arbor, MI 48109-2700 United States of America; 5 Department of Family Medicine, University of Michigan, 1018 Fuller SPC 5708, Ann Arbor, MI 48109 United States of America; 6 Phrachomklao Phetchaburi College of Nursing, 203 Tambon Thongchai, Mueang District, Phetchaburi Province 76000 Thailand; 7 Boromrajonani College of Nursing Nakon Lampang, 268 Pakham Road, Huawieng, Muang District, Lampang Province 52000 Thailand

**Keywords:** Non-communicable disease, intervention research, capacity-building, big data, Thailand

## Abstract

Thailand’s transition to high middle-income country status has been accompanied by demographic changes and associated shifts in the nation’s public health challenges. These changes have necessitated a significant shift in public health focus from the treatment of infectious diseases to the more expensive and protracted management of non-communicable diseases (NCDs) in older adults.

In 2010, in response to this shift in focus, the University of Michigan and colleagues at the Praboromarajchanok Institute for Health Workforce Development in Thailand began work on a broad-based multi-institutional programme for NCD research capacity-building in Thailand.

To begin to build a base of intervention research we paired our programme’s funded Thai postdoctoral fellows with United States mentors who have strong programmes of intervention research. One direct impact of the programme was the development of research ‘hubs’ focused upon similar areas of investigative focus such as self-management of cancer symptoms, self-management of HIV/AIDS and health technology information applications for use in community settings. Within these hubs, interventions with proven efficacy in the United States were used as a foundation for culturally relevant interventions in Thailand. The programme also aimed to develop the research support structures necessary within departments and colleges for grant writing and management, dissemination of new knowledge, and ethical conduct of human subject research.

In an effort to capitalise on large national health datasets and big data now available in Thailand, several of the programme’s postdoctoral fellows began projects that use data science methods to mine this asset. The investigators involved in these ground-breaking projects form the core of a network of research hubs that will be able to capitalise on the availability of lifespan health data from across Thailand and provide a robust working foundation for expansion of research using data science approaches.

Going forward, it is vitally important to leverage this groundwork in order to continue fostering rapid growth in NCD research and training as well as to capitalise upon these early gains to create a sustaining influence for Thailand to lead in NCD research, improve the health of its citizens, and provide ongoing leadership in Southeast Asia.

**Electronic supplementary material:**

The online version of this article (10.1186/s12961-019-0464-8) contains supplementary material, which is available to authorized users.

## Main Text

Thailand is now recognised by the World Bank as a high middle-income country [1]. As in many countries that undergo this transition, such economic progress has been sparked by rapid industrialisation and development accompanied by demographic changes and associated shifts in the nation’s public health challenges. In 2016, more than 11% of the Thai population (nearly 8 million people) were 65 years or older compared to just 5% in 1995 [2], giving Thailand the highest share of people aged over 65 of any developing nation in Asia [2]. This trend is set to continue, with Thailand’s elderly population projected to constitute more than a quarter of the population (some 17 million people) by 2040. This will result in significant undercutting in the age pyramid as birth and in-migration rates remain low [3]. Consequently, in the next decade, the number of persons over age 65 will outnumber those 15 and younger for the first time in Thai history [4–6], significantly skewing the Thai old-age dependency ratio from 12 people over age 65 per person of working age in 1997 to 16 in 2012 [5, 6]. These changes present substantial stressors to the Thai economy as increasing healthcare costs are borne by fewer and fewer working-age citizens. This is reflected in increased government spending on health, from 12% of total spending in 2000 to 16% in 2016. These changes have necessitated a significant shift in public health focus from the treatment of infectious diseases to the more expensive and protracted management of non-communicable diseases (NCDs) in older adults [5–7].

This epidemiologic trend was emerging in 2010 when the University of Michigan (UM) and colleagues at the Praboromarajchanok Institute for Health Workforce Development (PIHWD) in Thailand began work on building capacity for NCD research in Thailand. This objective aligned well with the fourfold mission of the PIHWD and the 40 health science colleges under its purview, namely (1) to produce and develop public health personnel; (2) to conduct research and develop knowledge; (3) the sharing of educational outcomes with other healthcare services; and (4) to reflect and maintain Thai culture and values. Comprehensive action would be required to reach the overarching goal for prevention and control of NCDs, i.e. a 2% per year reduction in death rates attributable to the main chronic diseases (heart disease, stroke, cancer, diabetes, respiratory diseases and dementias) [8]. To align with the Thai government’s two-pronged approach of (1) implementation of multi-sectoral policies aimed at decreasing population-level risks for NCDs and (2) effective and affordable delivery of health sector interventions for patients with NCDs, we recognised that a focus on evidence-based information was an essential foundation for both. However, evidence-based data requires culturally relevant research. Thus, the Thai government’s strategies rest on two important elements, namely on understanding population risk and age-related disease expression and on evidence-based interventions based on endemic research produced by a committed, local scientific community.

After an initial planning phase, we created a multi-faceted 5-year postdoctoral training programme to expand NCD research. The initial planning and subsequent training programme structure has been described elsewhere [9, 10]. In brief, the programme provided 2-year postdoctoral training in NCD research for 9 PhDs in nursing or related disciplines, 14 PhD-prepared investigators came to the University of Michigan for short-term training (1–2 months) related to NCDs, and 3 conferences were held in different regions of Thailand at which programme fellows and others presented NCD research findings and discussions were held on methods and means to enhance and expand research capacity for NCDs in Thailand. The purpose of this paper is to describe the impact of using a broad-based multi-institutional approach to building research capacity with an emphasis on methods that address the strategy of the Thai government by expanding evidence-based interventions and training in the use of country-level data resources to explicate population risk for and age-related disease expression of NCDs – both serving as the foundation for more rapid expansion of NCD research in Thailand.

### Using a broad-based multi-institutional approach

Whereas the often-used approach to building research capacity is to focus on deeply strengthening human and other resources within a single institution for a research focus area, we chose a broad-based multi-institutional approach to leverage several opportunities posed by the PIHWD and UM collaboration. This approach conforms to one of the basic principles of capacity development proposed by the United Nations Development Programme (UNDP) that “*emphasizes national systems, …*” and “*… discourages stand-alone project implementation units*” [11]. We incorporated several success factors addressed in the UNDP report as follows. First and foremost, PIHWD is a multi-institutional system of 40 colleges/universities and 62 hospital affiliations operating under the umbrella of the Thai Ministry of Public Health (MOPH). It provides administrative oversight and access to other MOPH structures and facilities that support research such as a growing Central Data Unit for country-level public health and clinical data. As such, PIHWD/MOPH has a well-developed organisation supporting research and translation to practice settings. The task of our collaboration was to use the existing and growing research resources to focus on NCD research in addition to the historically rich emphasis of the MOPH on prevention and effective intervention research for communicable diseases.

Second, the Thai government had already launched investments in developing country-level individual and public health datasets covering virtually all individuals in the country. The opportunity to focus emphasis on adding individual data that would track measures appropriate to NCDs, such as routine blood pressure, blood sugar, body weight, hospitalisation use for NCDs, etc., created new avenues for NCD research beyond those available in individual institutions. Third, the administrative leaders of the PIHWD were ready to embark on a system-level approach to expanding NCD research. Finally, the two emphasis areas for our research expansion for NCD, clinical intervention and data science studies, were not limited by the requirement of having well-developed individual institution structures for NCD research. This was because the central resources of PIHWD were accessible to the entire organisation and were tied administratively to the leaders of our PIHWD–UM partnership, thus making deployment of resources to the various settings across PIHWD possible.

### Expanding evidence-based intervention research

Based on a review of articles obtained from international and Thai journals, Thai NCD research has mainly involved surveys, followed by correlational, applied research including evidence-based practice, participatory research, health system research and clinical research [12, 13]. In terms of clinical research studies, the quasi-experimental design has most often been used, especially to test the effectiveness of case and care models. It is estimated that randomised controlled trial research represents less than 5% of all NCD studies. When compared to high-income countries, there is a relatively small number of large-scale NCD studies emanating from Thailand [14].

To begin to build a base of intervention research we paired our programme’s funded Thai postdoctoral fellows with United States mentors who have strong programmes of intervention research. During the ongoing programme seminar series, fellows, mentors and others were able to learn about the spectrum of intervention development from each other’s projects. In addition to the emphasis on the state of the science of a particular NCD, the seminars addressed a number of other key topics such as common methodologies, Institutional Review Board application, accruing a participant sample and developing collaborating partnerships in Thailand.

One direct impact of our efforts during the 5 years of the programme was the development of research ‘hubs’ focused upon similar areas of investigative focus such as self-management of cancer symptoms, self-management of HIV/AIDS and health technology information applications for use in community settings [15–19]. The development of a collaborative hub of investigators with like scientific foci required that each postdoctoral fellow began new collaborations upon their return to Thailand. Hub development also required the efforts of the programme leads to work with the Deans and Directors of research institutions where the postdoctoral fellows worked to further develop the overall research environment and to foster the collaborative linkages within and across institutions for similar scientific efforts. Because the PIHWD is a network of collaborating colleges and universities, this process of building linkages was more feasible than building bridges to institutions outside of the PIHWD. The rationale for this outcome is derived from our determination that PIHWD conformed to the characteristics necessary for effective capacity development as promulgated by the UNDP, i.e., that “*The way institutions/organizations are organized within the public sector architecture, their respective roles and coordination arrangements among those, how they manage human, physical and financial resources are all key determinants of organizational effectiveness*” [11]. For example, the PIHWD leads were able to periodically convene all Deans and Directors to discuss progress of the programme and capacity-building activities. The linkage to other public sector institutions was facilitated by involving key administrators from two such institutions as part of the capacity-building training programme, either as a direct consultant or as a member of the programme’s oversight committee.

Within these hubs, interventions with proven efficacy in the United States were used as a foundation for culturally relevant interventions in Thailand. In the cancer population, for example, the numbers of long-term survivors continue to increase and with this survivorship can come chronic symptoms that negatively impact health-related quality of life and use limited healthcare resources [15–17]. Fatigue, distressing menopausal symptoms, pain and peripheral neuropathy are all prevalent problems among cancer survivors. Thai nurse scientists have worked very successfully to validate important outcome measures in the Thai cancer survivor population and evaluate culturally transformed interventions for fatigue and menopausal symptoms. Similarly, intervention studies related to the management of HIV/AIDS symptoms as well as diabetes were completed in Thai samples to demonstrate efficacy as well as cultural and contextual relevancy in Thailand [15–20].

In addition to building these hubs, the programme aimed to develop the research support structures necessary within departments and colleges for grant writing and management, dissemination of new knowledge and ethical conduct of human subject research. The PIHWD universities and hospitals now each have institutional human subjects research boards with new experience in NCD research and early phase intervention studies. Future efforts will focus on building strong grant development and grants management support locally within university departments to keep pace with the volume and type of clinical intervention research to be expanded. Assisting researchers in finding sources for external funding of large NCD research projects is also an area for future work. Although there are several sources for funding NCD research at the national level in Thailand as well as internationally, it is difficult for new investigators to navigate the pathway to funding success. Additionally, greater emphasis on preparing investigators for grant writing and manuscript publication is needed to keep pace with research needs and opportunities.

As a consequence of the 5-year training and capacity-building programme there are now research hubs of investigators trained in clinical intervention research with experience in developing the foundational measurement development and pilot testing methods and interventions. Locations and more detailed descriptions of these hubs can be found in Additional file 1. There is a cross-hub network among the investigators that meets both virtually and at conferences, and there is a growing interest among collaborative scientists to engage in similar NCD research. The incentive to continue to deepen these research efforts stems from the Thailand public health strategic aims, which are now more clearly aligned with NCD research, prevention and early intervention [21] as well as the continued collaboration with strong NCD investigators at the UM.

Our future goals in furthering the development of NCD intervention research are to facilitate the development of large scale, multi-site intervention studies capitalising on the strengths of existing research hubs; foster the local training of investigators interested in NCD research through affiliation with existing hubs; develop new hubs that focus on additional PIHWD research priorities such as cardiovascular diseases and stroke; and further prepare PIHWD to lead the Southeast Asia Region in NCD research.

### Training to build data science capacity

The Thai government has recently invested in the development of national health surveys and clinical data capture using standardised measures along with the technology infrastructure that will support the analysis of both large datasets and big data across regions and provinces as a means of improving healthcare policy. The capacity to mine this data for research ranging from prevention strategies to disease treatments is emerging as a major asset of the health system in Thailand.

In an effort to build data science capacity, one of the programme mentors from the United States (an expert on the application of data mining methods to healthcare claims data) has given several presentations in Thailand related to the use of big data for healthcare research. Topics covered included mining health data, common sources of health/healthcare data, applications of big data analysis and Thailand’s national health data. Additionally, the Thai post-doctoral fellows were able to use the extensive data analytic platforms and methods available at the UM. Going forward it is a priority, and it will be critical to ongoing work, for the PIHWD and the MOPH to develop and support the analytic platforms and methods necessary for ‘big data’ in order to capitalise on this vital national resource for NCD research.

In an effort to extend the capacity for data mining in Thailand, several of the programme’s postdoctoral fellows began projects that use data science methods to mine currently available large national health datasets. Each project focused on a different NCD – dementia, diabetes mellitus (DM) or multiple chronic conditions (MCC). These fellows had little to no previous experience working with large datasets, but with the aid of their mentors and relevant courses, they were able to develop and carry out research projects using the rich regional and national data available in Thailand. For example, the ongoing dementia project aims to evaluate the robustness of current survey methods for identifying mild cognitive impairment (MCI) and dementia in older adults to improve diagnostic categorisation of people and to inform policy related to survey methods and measures. This objective will be accomplished by using data-merging methods across currently available large national health datasets, cross-sectional designs, statistical analyses, diagnostic categorisation of MCI and dementia in the Thai population, and techniques to identify individuals with MCI for early intervention. As part of that project, investigators are now analysing cross-sectional data on 174,227 adults over age 60 who reside in Udon Thani, Thailand, whose 2017 screening data on the national health survey include scores from the Abbreviated Mental Test and/or the Mini Mental Status Examination.

As a second example, the ongoing DM project is using data from the Thailand National Health Security Office to examine unplanned readmission rates of diabetes patients using over one million unique diabetes inpatients under the Universal Health Coverage Scheme. The study aims to determine the incidence and causes of 30-day readmission rates for patients with a primary and secondary diagnosis of DM and to develop an administrative claim-based algorithm for predicting 30-day readmission in the population. Additionally, the study aims to explore risk factors and causes of early admission using data mining approaches and to evaluate hospital performance using the Standardized Readmission Ratio with an approach adapted from the Hospital Readmission Reduction Program used by the Centers for Medicare and Medicaid Services, United States of America.

Third, the MCC project was one of the first to analyse the Thai National Health Dataset housed in the MOPH’s Health Information Technology and Communication Center. Specific aims were to (1) determine the extent to which the prevalence of adults with MCCs varies geographically and (2) examine variation in the annual cost of medications for adults with MCCs across the three national healthcare plans. This work led to the construction of national health datasets on the prevalence of NCDs across Thai districts and associated healthcare spending.

The investigators involved in these ground-breaking projects form the core of a network of research hubs that will be able to capitalise on the availability of lifespan health data from across Thailand and provide a robust working foundation for expansion of research using data science approaches.

### Going forward – leveraging our groundwork to accelerate growth in NCD research

During the last several years, our PIHWD–UM collaborative efforts have resulted in hubs of research expertise encompassing emerging intervention and data science approaches to NCD research, development of foundational institutional research support infrastructures for NCD research, and perhaps most importantly, it has influenced a national Thai policy emphasis on NCDs, including support of research expansion [21]. Our collaboration provides a framework for continued development of existing research hubs, expansion to other critical focus areas, institutional capacity expansion to support NCD research, and the advancement of research methodologies that have significant translational value for population health and chronic disease management. Our multi-institutional model of research capacity-building may also be useful in countries now experiencing a similar rise in NCDs that accompanies similar demographic and economic transitions.

## Conclusion

It is now vitally important to leverage this foundation in order to continue fostering rapid growth in NCD research and training, and to capitalise upon these early gains to create a sustaining influence so that Thailand can lead NCD research, improve the health of its citizens, and provide ongoing leadership in the Southeast Asia Region. For its part, the PIHWD has committed to achieve this growth through the creation of a national centre for NCD study that will address the emerging needs of the PIHWD institutions and research hubs in furthering their NCD research efforts.

## Additional file

Additional file 1: Research Hubs. Location and description of research hubs arising from the con-communicable diseases training programme. (DOCX 172 kb)

## Data Availability

Not applicable.

## Abbreviations

DM: Diabetes mellitus
MCC: Multiple chronic conditions
MCI: mild cognitive impairment
MOPH: Ministry of Public Health
NCD: Non-Communicable Disease
PIHWD: Praboromarajchanok Institute for Health Workforce Development
UM: University of Michigan
UNDP: United Nations Development Programme
