# Supplementary material for: Impact of using a broad-based multi-institutional approach to build capacity for non-communicable disease research in Thailand
Source: Health Res Policy Syst. 2019 Jun 14;17:62. doi: 10.1186/s12961-019-0464-8 (PMC6570856; doi:10.1186/s12961-019-0464-8)
Supplement: Supplementary file 1 — Research Hubs. Location and description of research hubs arising from the con-communicable diseases training programme. (DOCX 172 kb) [file 12961_2019_464_MOESM1_ESM.docx]

**Research Hubs**


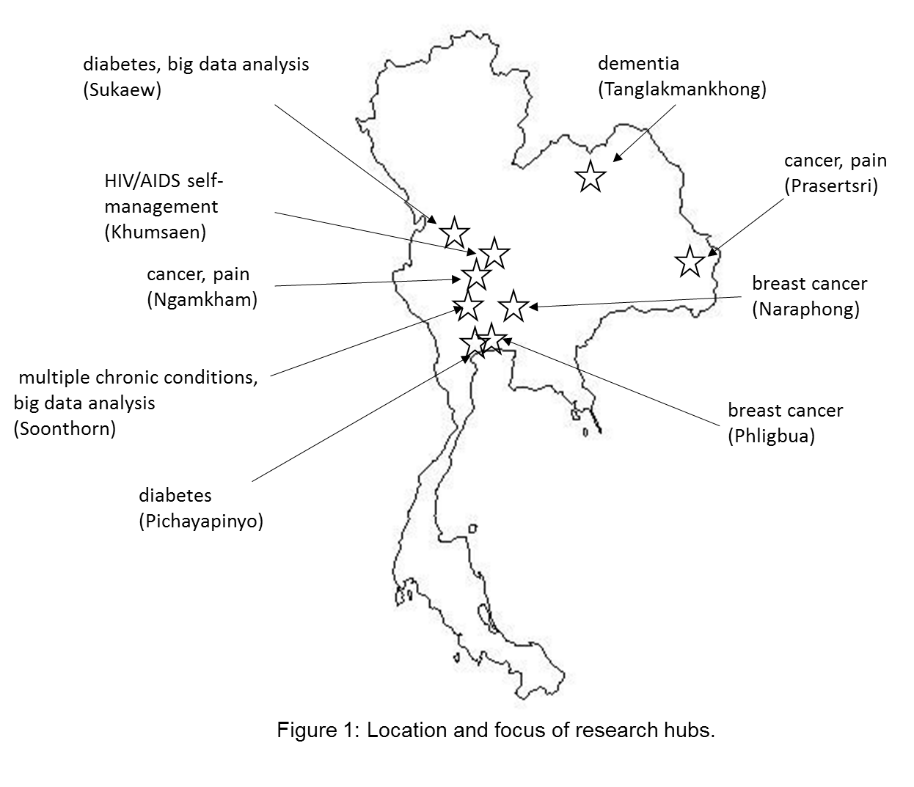
The research ‘hubs’ are informally comprised of trainees, mentors and collaborators focusing on research in similar NCD diseases, symptoms or treatment methods (Figure 1). The hubs are maintained by formal and informal meetings through virtual communication platforms to share research approaches. Most were initially formed by trainees having a common UM mentor or collaborating mentor. Other hubs have formed with collaborators around big data sources for dementia, diabetes mellitus (DM) and multiple chronic conditions (MCC). The specific research accomplished by each of these D43 fellows and their U.S. and Thai collaborators are described below.

**Dr. Thitiporn Sukaew**. Dr. Sukaew’s research focused on diabetes mellitus and the potential of using the rich national data made available through the National Health Security Office (NHSO), see section G. subsection Data Science Approaches for NCD Research. With UMSN mentor Dr. Ivo Dinov, Dr. Sukaew has accquired the unique skills and capacity to manipulate, process, analize and visualize complex heterogeneous data archives. Dr. Sukaew and Dr. Dinov conducted a study to examine unplanned readmission rate of diabetes patients using over 1 million unique diabetes inpatients under the Univeral Health Coverage Scheme (UHCS). The study aimed to determine the incidence and causes of 30-day readmission rates for patients with a primary and secondary diagnosis of DM and to develop an administrative claim-based algorithm for predicting 30-day readmission in the population. Additionally, the study aims explored risk factors and causes of early admission using data mining approaches, and evaluated hospital performance using the Standardized Readmission Ratio with an approach adapted from the hospital readmission reduction program (HRRP) by the Centers for Medicare and Medicaid Services, USA. The results suggest a significant relationship between the rate of hospital readmission cost and readmission rate for the top 25 high-volume health conditions in the Thai database especially for three important disease cohorts (IDC-10 codes): heart failure (I50), angina pectoris (I20) and COPD (J44). The next step will be to develop a diabetes data warehouse with over 3 million unique diabetes patients under UHCS which covers over 32 million out-patient hospital visits during 2011-2015. They expect to extract an underlying pattern of risk factors associated with diabetes incidence and progression using a combination of conventional statistical methods and machine learning approach.

**Dr. Surasak Soonthorn**. Dr. Soonthorn’s research examined how the cost of medications for adults with MCCs differed across the three national health care plans. With UMSN mentor Dr. Matthew Davis, Dr. Soonthorn gained important skills in the use of a unique combination of concepts and methods from epidemiology, health services research, health economics, and geographic information systems (GIS) to test the central hypothesis that there exists unrecognized variation in the cost of prescription medication for the population of adult Thais with MCCs. The project was one of the first to analyze the Thai National Health Data set housed in the MOPH Health Information Technology and Communication Center. Specific aims were to 1) Determine the extent to which the prevalence of adults with MCCs varies geographically, and 2) Examine variation in the annual cost of medications for adults with multiple chronic conditions across the three national health care plans. Dr. Soonthorn has constructed national health datasets on the prevalence of NCDs across Thai districts and associated healthcare spending. Using national data on more than 34 million adult Thais, Dr. Soonthorn identified 3.9 million with hypertension, 2.5 million with diabetes, 0.8 million with cancer, 0.7 million with chronic respiratory disease, 0.3 million who suffered a stroke, and 0.8 million with other cardiovascular disease in 2016. Dr. Soonthorn’s work has been among the first to demonstrate significant regional variation in NCDs – the MCC population varied from more than 200 per 1,000 to 4 per 1,000 and the median age- and sex- adjusted prevalence was 25.4 per 1,000 (IQR: 18.9 to 33.9). His future work will examine the effect of a particular health insurance plan on prescription medication spending among adults with MCCs (policies regarding coverage of brand versus generic medications).

**Dr. Kamonthip Tanglakmankhong**. Dr. Tanglakmankhong’s focus is to evaluate the robustness of current survey methods for identifying mild cognitive impairment (MCI) and dementia in older adults to improve diagnostic categorization of people and to inform policy related to survey methods and measures. Working with Dr. Kathleen Potempa (UMSN) and Dr. Ben Hampstead (UM Psychiatry), Dr. Tanglakmankhong’s training includes data merging methods across currently available large national health data sets, cross-sectional designs, statistical analyses, diagnostic categorization of MCI and dementia in the Thai population, and techniques to identify individuals with MCI for early intervention. Under analysis now is a cross-sectional study of 174,227 adults over age 60 who reside in Udon Thani, Thailand whose 2017 screening data on the national health survey included measurement of the Abbreviated Mental Test (AMT) and the Mini Mental Status Examination (MMSE-Thai). Specific aims are to: 1) Compare the distribution of AMT scores to determine the appropriate delineation for MCI; 2) Evaluate the comparative validity of the AMT and MMSE-Thai for determining MCI and/or dementia; 3) Describe the distribution of scores of both AMT and MMSE-Thai, adjusted for age, education and sex, to provide normative reference for assessing cognitive decline in Thai people.

**Dr. Srisuda Ngamkham**. Dr. Ngamkham’s interest is in pain management in people with cancer. Working with UMSN mentor Dr. Ellen Smith, Dr. Ngamkham is gaining valuable skills in adapting evidence-based interventions to treat pain in cancer patients, adapting culturally appropriate methods in RCT development, including cultural and language validation of measures, to the Thai population, and statistical methods and procedures. Their current study focuses on the development of an RCT study of Thai Buddhism-based Mindfulness (TBbM) for Pain Management in Thai Outpatients with Cancer. The aims of their ongoing study are: 1) Compare the effect of the TBbM intervention to that of usual care on worst pain severity; 2) Compare the effect of the TBbM intervention to that of usual care on secondary outcomes (i.e., pain interference, average pain, anxiety and depression, mindfulness, locus of control, and QoL); and 3) Explore the mediating effects of TBbM-induced changes in locus of control and anxiety and depression on worst pain severity. The investigators posit that the TBbM intervention will be more effective than usual care in reducing pain (primary outcome) and improving pain interference, anxiety and depression, mindfulness, locus of control, and QoL (secondary outcomes).

**Dr. Wipasiri Naraphong**. Dr. Naraphong’s research focus is on the use of self-management therapies in cancer patients. Working with UMSN mentor, Dr. Debra Barton and in collaboration with Dr. Andrea Barsevick at the Thomas Jefferson University – Dr. Naraphong developed a self-management energy conservation program for cancer-related fatigue in Thai women with breast cancer receiving chemotherapy. Dr. Naraphong has gained important skills in evidence-based RCT development, cognitive-behavioral therapy techniques, and cultural adaptation of research to the context of Thailand. This is the first pilot study to test the preliminary effects of an energy conservation intervention that utilizes effective principles of cognitive-behavioral therapy and cultural diversity between Thailand and the US to decrease fatigue severity. The purpose of this pilot study was to preliminarily examine a 12-week self-management energy conservation program (ECAM)’s effects on fatigue, and secondarily on sleep, anxiety and depression, and physical activity in Thai women with breast cancer undergoing chemotherapy. The study was completed in May 2018 and Dr. Naraphong is in the process of planning a large-scale clinical trial.

**Dr. Panan Pichayapinyo**. Dr. Pichayapinyo’s interest is in the use of electronic, mobile methods for better self-management of people with diabetes mellitus. Dr. Pichayapinyo worked with Drs. Laura Saslow (UMSN) and James Aiken (UM Diabetes Center) and gained skill in RCT research development, evidence based self-management interventions for DM, use of mHealth methodologies, and cultural adaptation of evidence-based interventions. Her preliminary work was to study the adaptation, usability, and feasibility of a mobile health (mHealth) system to improve type 2 diabetes self-management in Thailand – a culturally appropriate modification of the system used in the U.S. This study aims to: 1) investigate the feasibility and acceptability of a culturally- and clinically-adapted mHealth intervention with adult Thai diabetic patients and their community nurses, and 2) estimate the intervention’s effect upon glycated hemoglobin (HbA1c), fasting blood glucose, self-management behaviors, and diabetes-related distress in uncontrolled type 2 diabetes. The results indicated that mean HbA1c decreased by 0.9% after intervention. Patients reported health behavior changes including reduced carbohydrate consumption, increased physical activity, increased medical adherence, reduced sleep disturbance, and more frequent foot care. Both patients and nurses would recommend the intervention to others, but nurses had some concerns regarding increased work burden. Dr. Pichayapinyo along with UM (Saslow and Aiken) and Thai collaborators (Rawdaree) are preparing an R21 submission for a larger RCT trial of the mHealth system.

**Dr. Natawan Khumsaen**. Dr. Khumsaen’s interest is in the prevention and management of HIV as a chronic disease. Dr. Khumsaen and her mentor Dr. Stephenson (UMSN) adapted the HIV/AIDS self-management education program ADAPT-ITT framework for Men who have Sex with Men (MSM) for use in Thailand. Dr. Khumsaen gained skill in adaptation of evidence based self-management programs for HIV prevention in Thailand, designing RCT, and building collaborative teams for ongoing research. The ADAPT-ITT framework consists of 8 phases and during two years as a postdoc Dr. Khumsaen finished phase 1-7 of the ADAPT-ITT – the first time this framework and related studies of each phase were used in MSM Thai population. Currently Drs. Khumsaen, Stephenson and a new collaborator Dr. Yongyuth Watthanachai, who leads a healthcare team at the HIV clinic in Sappasithiprasong (SPS) hospital, Ubonratchathanie (Ubon) province, Thailand, are resubmitting a scored NIH R21 application for a randomized control trial (RCT) to test the efficacy of the HASMEP-Thai – an intervention proved effective in the U.S. – among HIV-positive Thai MSM.

**Dr. Warunee Phligbua**. Dr. Phligbua is interested in self-management therapies for women with breast cancer. Working with Dr. Debra Barton (UMSN) and Dr. Ellen Smith (UMSN), Dr.Phligbua has been trained in hypnotherapy by Dr. Barton and Dr. Gary Elkins from the Baylor University. She has also gained skills in the development of evidence-based RCT, application of interventions used in U.S. population to Thailand adjusting for cultural and contextual differences, design and methodology, and statistical analyses. Dr. Phligbua developed and tested the use of hypnosis in the management of menopausal symptoms in Thai women with a history of breast cancer. Dr. Phligbua’s two-phase study addressed both the measurement applications and intervention for hot flash relief. Phase I evaluated the psychometric properties of the Menopause-Specific Quality of Life (MENQOL) scale. Phase II evaluated the feasibility and effect of hypnosis versus a white noise control on hot flashes in women with a history of breast cancer. Dr. Phligbua completed both phases in 2017. She is in the process of developing a large scale RCT for this intervention and is making application for R21 funding as well as funding from Thailand.

**Dr. Nusara Prasertsri**. Dr. Prasertsri’s primary interest is in understanding the incidence of chemotherapy-induced peripheral neuropathy (CIPN) in patients undergoing cancer treatment in Thailand. Dr. Ellen Smith (UMSN) was the primary mentor for Dr. Nusara Prasertsri forming a collaboration to determine the cultural and clinical relevance of CIPN in patients undergoing cancer treatment in Thailand. While Thai patients reported CIPN during/after neurotoxic chemotherapies, CIPN was not routinely measured or treated in most clinical settings in Thailand. Prasertsri and Smith determined that the underlying issue was inadequate knowledge of CIPN causes, symptoms and treatments. Prasertsri and Smith created a mobile application called mCIPN to be used by nurses in the clinical setting that provided information about assessment, symptoms and clinical management of CIPN. Prasertsri and Smith then designed and conducted a prospective study to test the feasibility and efficacy of the mCIPN with nurses caring for people with CIPN. The study used implementation science methodologies to evaluate the feasibility, usability, acceptability, and satisfaction of nurse-focused CIPN teaching approach. The study is completed and a large scale RCT is now being planned.
